# A transient CRISPR/Cas9 expression system for genome editing in *Trypanosoma brucei*

**DOI:** 10.1186/s13104-020-05089-z

**Published:** 2020-06-03

**Authors:** Sebastian Shaw, Sebastian Knüsel, Sarah Hoenner, Isabel Roditi

**Affiliations:** 1 Institute of Cell Biology, University of Bern, Bern, Switzerland; 2 Graduate School of Cellular and Biomedical Science, University of Bern, Bern, Switzerland; 3 Present Address: Department of Pathobiology, School of Veterinary Medicine, University of Pennsylvania, 380 South University Avenue, Philadelphia, PA 19104 USA

**Keywords:** CRISPR/Cas9, Transient transfection, Trypanosome

## Abstract

**Objective:**

Generation of knockouts and in situ tagging of genes in *Trypanosoma brucei* has been greatly facilitated by using CRISPR/Cas9 as a genome editing tool. To date, this has entailed using a limited number of cell lines that are stably transformed to express Cas9 and T7 RNA polymerase (T7RNAP). It would be desirable, however, to be able to use CRISPR/Cas9 for any trypanosome cell line.

**Results:**

We describe a sequential transfection expression system that enables transient expression of the two proteins, followed by delivery of PCR products for gRNAs and repair templates. This procedure can be used for genome editing without the need for stable integration of the Cas9 and T7RNAP genes.

## Introduction

**Fig. 1:**
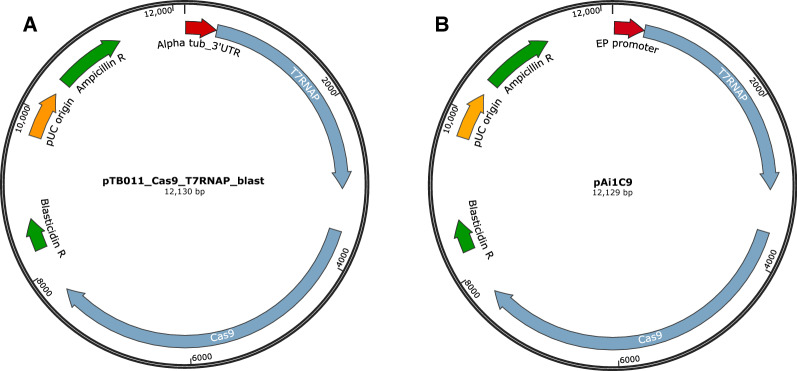
Plasmid maps of **a** pTB011_Cas9_T7RNAP_blast and **b** pAi1C9

The establishment of genome editing by CRISPR/Cas9 in trypanosomatids has greatly increased the ease with which knockouts can be generated, as two copies of a non-essential gene can often be deleted in a single round of transfection. The system most widely used for *Trypanosoma brucei* entails creating cell lines which express (either constitutively or inducibly) both Cas9 to generate a DNA double-strand break and T7RNAP for the transcription of guide RNAs from a DNA template [1–4]. The original pTB011 plasmid generated for *T. brucei* [4] encodes Cas9 flanked by tubulin sequences, enabling the construct to be integrated into the corresponding multicopy array. We modified this plasmid by replacing the puromycin acetyltransferase gene with that of T7RNAP, giving rise to pTB011_Cas9_T7RNAP_blast (Fig. 1a). This plasmid allows generation of CRISPR/Cas9-competent cell lines, constitutively expressing T7RNAP and Cas9 from the tubulin locus, in a single round of transfection [5]. Further modification of this plasmid, and optimisation of the transfection protocol for transient expression of the proteins and guide RNAs, are described below.

## Main text

### Results

In order to expand the range of cell lines that can be genetically modified by CRISPR/Cas9, without the need for stable transformation, we developed a protocol for transient expression of all three components (Cas9, T7RNAP and guide RNAs) in *T. brucei*. The plasmid All-in-one-Cas9 (pAi1C9; Fig. 1b) was constructed by replacing the α-tubulin sequence upstream of T7RNAP with 366 bp of the EP procyclin promoter and 5′ UTR [6].

**Fig. 2:**
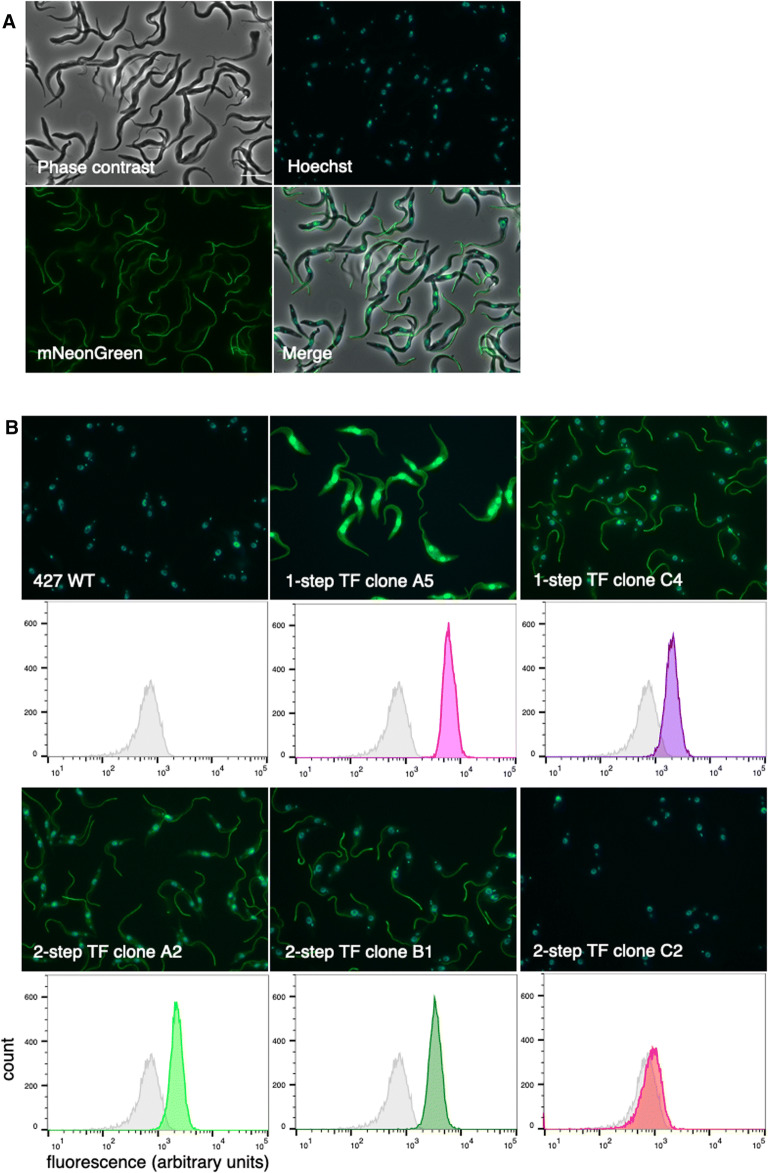
Trypanosomes tagged with mNeonGreen at the C-terminus of phosphodiesterase B1 (PDEB1). **a** Tagging PDEB1 in Lister 427/Cas9. Scale bar: 10 microns. **b** Tagging PDEB1 in Lister 427. Fluorescence intensity of live cells was quantified with a benchtop flow cytometer (ACEA NovoCyte). To remove particles of subcellular size, a cut-off of 3 x 10^4^ was applied to the forward scatter. A total of 10^4^ events were recorded and analysed using FlowJo software without gating. TF transfection

To test the functionality of the plasmid, we used the LeishGEdit software [4] to design a guide RNA that allows tagging of the C-terminus of phosphodiesterase B1 (PDEB1) with mNeonGreen (mNG). The repair template, in the form of a purified PCR product, including homology arms of 30 bp on each side, was amplified from pPOTv7 [7]. As a positive control we transfected a derivative of *T. brucei* Lister 427 procyclic forms that already expresses Cas9 and T7RNAP constitutively (Lister 427/Cas9) [5]. This gave rise to cells with a fluorescent flagellum as expected (tryptag.org) (Fig. 2a).

For genome editing by transient expression of Cas9 and T7RNAP, we tested two different transfection schemes. Initially, all components were transfected simultaneously: the circular plasmid pAi1C9 encoding Cas9 and T7RNAP, the DNA template for in vivo transcription of a guide RNA, and the DNA repair template harbouring mNG for C-terminal tagging and a hygromycin resistance gene. This procedure gave few hygromycin-resistant clones, only 10 clones in total, from 4 separate transfections. These clones either did not express mNG (4 clones), or expressed it as a cytoplasmic protein (2 clones) or expressed it correctly localised to the flagellum (4 clones). Examples are shown in Fig. 2b. We hypothesise that, due to insufficient expression of Cas9 and T7RNAP, the amounts of site-specific guide RNAs and double-strand breaks at the correct locus were too low to drive efficient integration by homology-directed repair.

**Fig. 3:**
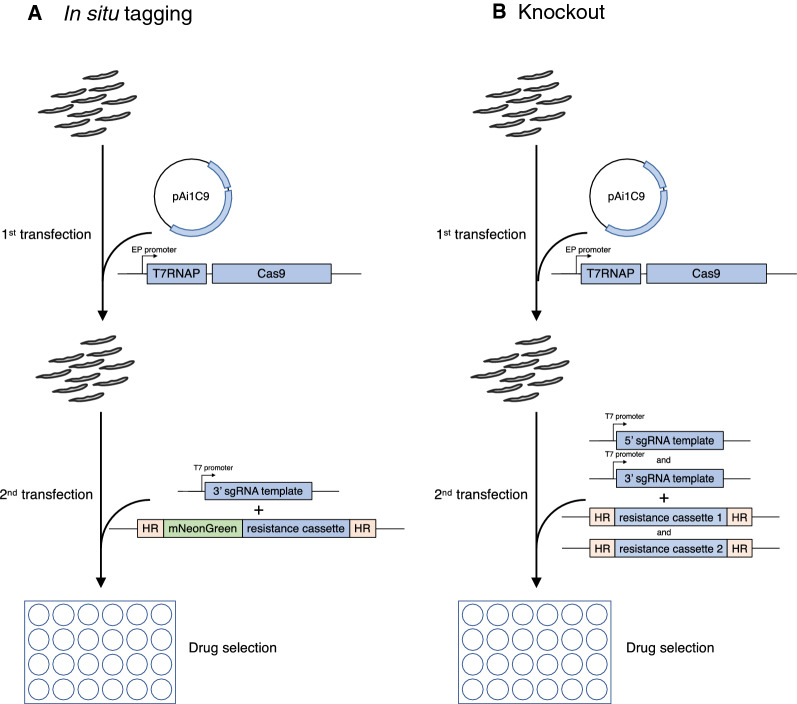
Flow chart of sequential transfections. Procyclic form trypanosomes were transfected first with pAi1C9 to allow expression of Cas9 and T7RNAP. After 20 h, the pool of trypanosomes was transfected a second time to provide the templates for gRNA(s) and the repair template(s)/resistance cassette(s)

We therefore tested sequential transfections in which we first used pAi1C9 to enable expression of Cas9 and T7RNAP. A second transfection 20 h later provided the templates for the gRNA and the mNG repair construct. The same electroporation conditions were used for the plasmid and the templates (see Methods and Fig. 3). This procedure yielded 20 clones. There were 16 clones in which mNG was correctly localised to the flagellum, one in which it was cytoplasmic, 2 clones with mixed populations of mNG localised either to the flagellum or the cytoplasm, and one that was negative (see Fig. 2b for representative examples). One clone with flagellar mNeonGreen had a fluorescence intensity twice that of the others (clone B1) and might have been tagged on both alleles.

We have also used this procedure of sequential transfections to simultaneously knock out both copies of trypanin or GPI-anchor transamidase subunit 8 (GPI8), which are non-essential genes in cultured procyclic form *T. brucei* [8, 9]. Genotyping data is provided in Additional file 1.

## Methods

### Transfection of pAi1C9

4 × 10^7^*T. b. brucei* 427 procyclic forms were transfected with 10 µg pAi1C9 dissolved in 100 µl TbBSF transfection buffer [10], using an Amaxa Nucleofector IIb (Lonza), program X-014 [11]. Cells were transferred to 13 ml SDM79 medium [12] supplemented with 10% FBS and incubated at 27 °C and 2.5% CO_2_ for 20 h.

### Transfection of pooled PCR products for repair constructs and sgRNA templates

The entire culture from the first transfection was centrifuged at 1700 g for 5 min, the supernatant discarded and the cells resuspended in 100 µl TbBSF transfection buffer containing the pooled PCR products (see protocol for PCRs below). For tagging, one sgRNA template (targeted to the 3′ end of the ORF) and one repair template (hygromycin resistance) were provided; to generate the knockouts, we provided two sgRNA templates (targeted to the 5′ and 3′ ends of the ORF) and two repair templates (hygromycin and neomycin resistance genes).

Transfection was performed as described above and the cells were transferred to 10 ml SDM79 supplemented with 10% FBS. The cells were diluted 1:5, 1:50 and 1:500 in conditioned medium (fresh medium + 20% supernatant of a log-phase culture) and distributed into 24-well plates (1 ml in each well). Transformants were selected using 25  µg  ml^−1^ Hygromycin B and/or 15  µg  ml^−1^ Geneticin; stable clones were obtained 2 weeks post selection.

### Polymerase chain reactions (PCR)

Reactions were performed with reagents from New England Biolabs: Phusion High-Fidelity DNA polymerase (M0530S), 5 x  Phusion HF buffer (B0519S) and dNTP mix (N0447S). Cycling conditions are identical to those previously published [4, 13].

- 1st PCR: template for sgRNAs; two 20 µl reactions per target.
  - 1 μl G00 primer (0.5 μM)
  - 4 μl 5 x Phusion HF buffer
  - 0.5 μl dNTP mix (250 μM)
  - 1 μl sgRNA primer (0.5 μM)
  - 0.2 μl Phusion High-Fidelity DNA polymerase (0.4U)
  - 13.3 μl H_2_O
- Program:
  1. 30′′, 98 °C
  2. 10′′, 98 °C
  3. 30′′, 60 °C
  4. 15′′, 72 °C
  5. go to step 2, 35 cycles in total
  6. 10′, 72 °C
  7. hold at 10 °C
- 2nd PCR: template for resistance gene; two 40 µl reactions per resistance gene
  - 2 μl 60 ng pPOTv7 mNG (hygro or G418) plasmid
  - 8 μl 5 x Phusion HF buffer
  - 1 μl dNTP mix (250 μM)
  - 2 μl Upstream forward primer (0.5 μM)
  - 2 μl Downstream reverse KO/TAG primer (0.5 μM)
  - 0.4 μl Phusion High-Fidelity DNA polymerase (0.4U)
  - 24.6 μl H_2_O
- Program:
  1. 5′, 94 °C
  2. 30′′, 94 °C
  3. 30′′, 65 °C
  4. 2′30′′, 72 °C
  5. go to step 2, 40 cycles in total
  6. 10′, 72 °C
  7. hold at 10 °C

### DNA purification after PCR

PCR reactions were pooled and extracted with 1 volume water-saturated phenol (pH 8), followed by extraction with 1 volume chloroform. DNA was precipitated from the aqueous phase by addition of 0.1 volume 3 M sodium acetate, pH 5.2, and 3 volumes ice-cold ethanol. The DNA was pelleted by centrifugation, washed twice with 1 ml 80% ethanol, air-dried at room temperature, dissolved in 40 μl Milli-Q-water and stored at − 20 °C until transfection.

### Primers

G00: aaaagcaccgactcggtgccactttttcaagttgataacggactagccttattttaacttgctatttctagctctaaaac

PDEB1 3′ sgRNA primer:

gaaattaatacgactcactataggTGAAGAAGTCAGTTGACCGGgttttagagctagaaatagc

Trypanin 5′ sgRNA primer:

gaaattaatacgactcactataggCAAAAACGAGAAGAGCCTACgttttagagctagaaatagc

Trypanin 3′ sgRNA primer:

gaaattaatacgactcactataggAGGTGTTGTGGTTCACACGTgttttagagctagaaatagc

GPI8 5′ sgRNA primer:

gaaattaatacgactcactataggCGGTTGCAAAAAACGAATGCgttttagagctagaaatagc

GPI8 3′ sgRNA primer:

gaaattaatacgactcactataggGGTATGTCCCATCAGTTGGAgttttagagctagaaatagc

Trypanin upstream forward primer:

TACTTTTCAGACTGCATCGTGGCGTACCCCgtataatgcagacctgctgc

Trypanin downstream reverse KO primer:

CTGCAACAAAGCCGTAACTTGGAACAACCAccggaaccactaccagaacc

PDEB1 downstream forward primer:

ACGAGTTCTGGCAACAACAGCAGTACTCGTggttctggtagtggttccgg

PDEB1 downstream reverse TAG:

ATCACCATTGACAAGAACGTACATCTACCAccaatttgagagacctgtgc

GPI8 upstream forward primer:

TTGGATCAGGCGCTTGCATATTTATTTCCAgtataatgcagacctgctgc

GPI8 downstream reverse KO primer:

AGTTTCAGGAAGGAAGTTCGTTTTTCTCCTccggaaccactaccagaacc

## Limitations

Transient transfection with pAi1C9 gives rise to fewer clones than cell lines that are stably transformed with the T7RNAP and Cas9 genes. It has the advantage, however, that it can be applied to any *T. brucei* cell line. Moreover, in contrast to stable integration of the CRISPR/Cas9 machinery, transient transfection does not require additional selectable markers and it has the added advantage that it circumvents possible Cas9 toxicity. In addition to using Cas9 for deletion, mutation, tagging or integration of ectopic copies, this procedure could also be adapted for nuclease inactive Cas9 variants for targeting RNAs or epigenetic modifications [14, 15].

## Supplementary information

**Additional file 1:** Validation of knockout (KO) clones. **A+B** Trypanin-KO (Tb427.10.6350). **A** Clonal selection of stable transformants in 24-well plates. Dilutions from transfected pool cultures and number of cells seeded are indicated. Circles depict wells containing cells with successful (green filled circle) or unsuccessful (○) genome editing. **B** Assessment of editing by genotyping PCR and agarose gel electrophoresis. Genes amplified from genomic DNA and target loci are indicated. Lanes corresponding to validated KO clones are marked with green circles. **C+D** GPI8-KO (Tb427.10.13860) in a cell line allowing inducible expression of an ectopic copy of GPI8. **C** as in **A**. **D** as in **B**. **E** List of primers used for genotyping PCRs of Trypanin-KO (left panels) and GPI8-KO (right panels).

## Data Availability

Plasmids are available on request from Isabel Roditi (Isabel.roditi@izb.unibe.ch). The nucleotide sequences of pTB011_Cas9_T7RNAP_blast and pAi1C9 are provided on this website: https://www.izb.unibe.ch/research/prof_dr_isabel_roditi/index_eng.html.

## Abbreviations

mNG: mNeonGreen
PDEB1: Phosphodiesterase B1
T7RNAP: T7 RNA polymerase
