# Supplementary material for: A transient CRISPR/Cas9 expression system for genome editing in Trypanosoma brucei
Source: BMC Res Notes. 2020 Jun 3;13:268. doi: 10.1186/s13104-020-05089-z (PMC7268226; doi:10.1186/s13104-020-05089-z)
Supplement: Supplementary file 1 — Additional file 1: Validation of knockout (KO) clones. A+B Trypanin-KO (Tb427.10.6350). A Clonal selection of stable transformants in 24-well plates. Dilutions from transfected pool cultures and number of cells seeded are indicated. Circles depict wells containing cells with successful (green filled circle) or unsuccessful (○) genome editing. B Assessment of editing by genotyping PCR and agarose gel electrophoresis. Genes amplified from genomic DNA and target loci are indicated. Lanes corresponding to validated KO clones are marked with green circles. C+D GPI8-KO (Tb427.10.13860) in a cell line allowing inducible expression of an ectopic copy of GPI8. C as in A. D as in B. E List of primers used for genotyping PCRs of Trypanin-KO (left panels) and GPI8-KO (right panels). [file 13104_2020_5089_MOESM1_ESM.pdf]

## Additional File 1: Validation of knockout clones

### A Trypanin-KO – clonal selection in 24 well plates

Number of successfully edited clones (or mixed populations):
**Full KO: 3 of 13 tested (● of ○)**

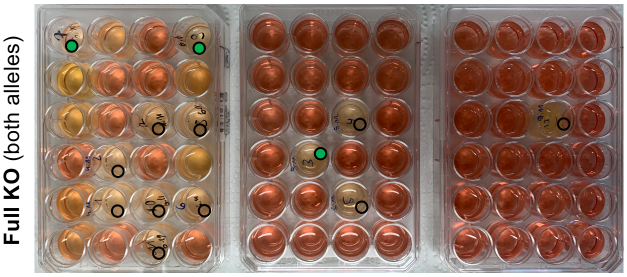


### B Trypanin-KO – PCR genotyping

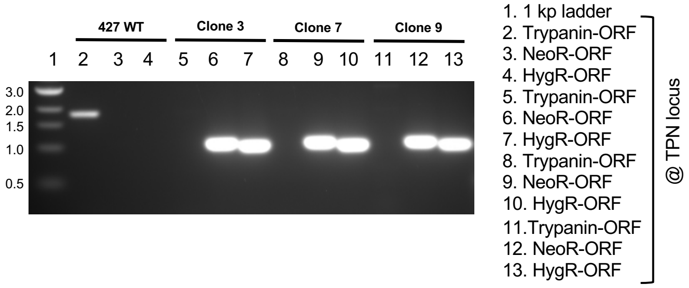


### C GPI8-KO – clonal selection in 24 well plates

Number of successfully edited clones (or mixed populations):
**Full KO: 1 of 23 tested (● of ○)**

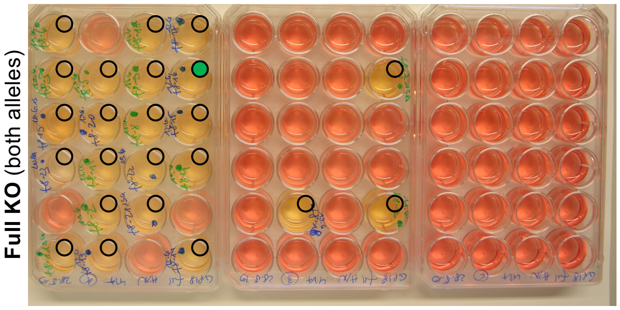


Number of successfully edited clones (or mixed populations):
**Semi KO: 11 of 12 tested (● of ○)**

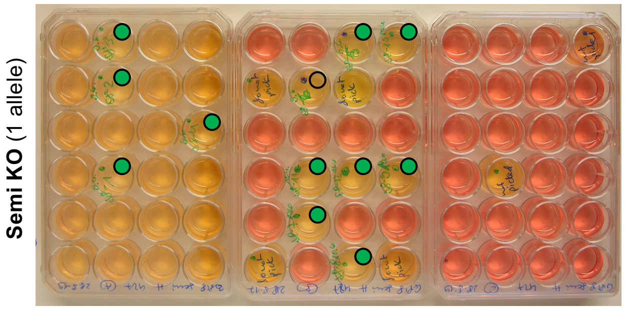


### D GPI8-KO – PCR genotyping

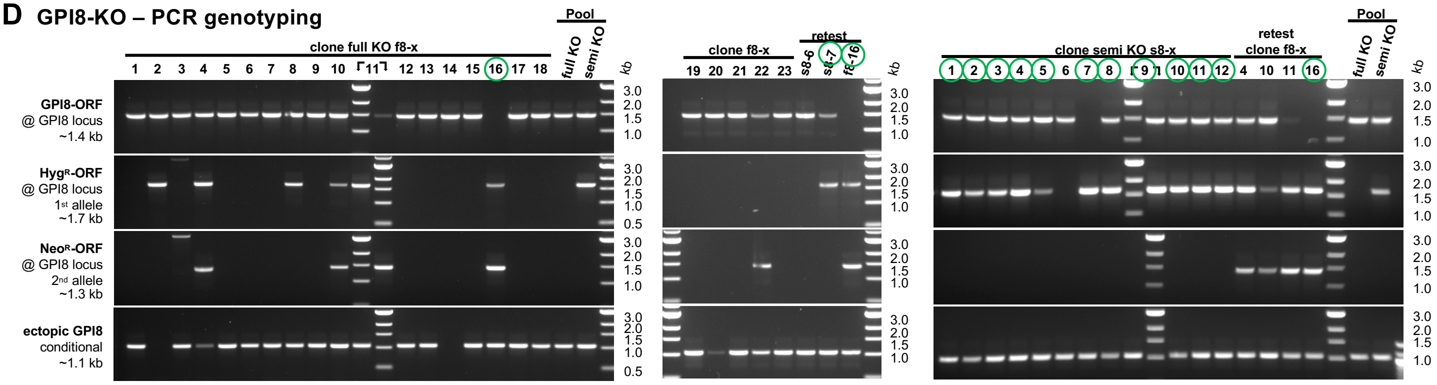


### E Primer sequences for genotyping PCR

**Trypanin-KO – primers used:**

**Trypanin-ORF @ TPN locus:**
Tryp-5Flk_Fw + Tryp-ORF_Rv

**Hyg$^R$-ORF @ TPN locus:**
Tryp-5Flk_Fw + R2_Hyg-

**Neo$^R$-ORF @ TPN locus:**
Tryp-5Flk_Fw + R3_Neo-

**Trypanin-KO – primer sequences (5'-3'):**

**Tryp-5Flk_Fw:**
GCTGAGATAGTTTAAGAGGGAGAG
**Tryp-ORF_Rv:**
GACATATGCTACTCAAAGTTGCTCCGTG
**R2_Hyg-:**
TACACATGGGGATCAGCAAT
**R3_Neo-:**
TCGATGCGATGTTTCGCTTG

**GPI8-KO – primers used:**

**GPI8-ORF @ GPI8 locus:**
5Flk-GPI8_Fw + GPI8-ORF_Rv

**Hyg$^R$-ORF @ GPI8 locus:**
5Flk-GPI8_Fw + Hygro_Rv

**Neo$^R$-ORF @ GPI8 locus:**
5Flk-GPI8_Fw + Neo_Rv

**ectopic GPI8, conditional copy:**
SPU + GPI8-ORF_Rv

**GPI8-KO – primer sequences (5'-3'):**

**5Flk-GPI8_Fw:**
AGCATTGGTGGCAATATC
**GPI8-ORF_Rv:**
GTCATATGCTAGAACAAATCGTAACG
**Hygro_Rv:**
gtaatatcgatCTATTCCTTTGCCCTCGGAC
**Neo_Rv:**
ataccatggTCAGAAGAACTCGTCAAGAAG
**SPU:**
GCTGCACGCGCCTTCGAGTT

**Additional File 1 – Validation of knockout (KO) clones. A+B** Trypanin-KO (Tb427.10.6350). **A** Clonal selection of stable transformants in 24-well plates. Dilutions from transfected pool cultures and number of cells seeded are indicated. Circles depict wells containing cells with successful (●) or unsuccessful (○) genome editing. **B** Assessment of editing by genotyping PCR and agarose gel electrophoresis. Genes amplified from genomic DNA and target loci are indicated. Lanes corresponding to validated KO clones are marked with green circles. **C+D** GPI8-KO (Tb427.10.13860) in a cell line allowing inducible expression of an ectopic copy of GPI8. **C** as in **A**. **D** as in **B**. **E** List of primers used for genotyping PCRs of Trypanin-KO (left panels) and GPI8-KO (right panels).
